# Methyl Gallate Improves Hyperuricemia Nephropathy Mice Through Inhibiting NLRP3 Pathway

**DOI:** 10.3389/fphar.2021.759040

**Published:** 2021-12-20

**Authors:** Peng Liu, Wen Wang, Qiang Li, Xin Hu, Bingyong Xu, Chen Wu, Lijie Bai, Li Ping, Zhou Lan, Lvyi Chen

**Affiliations:** ^1^ School of Pharmaceutical Sciences, South-Central University for Nationalities, Wuhan, China; ^2^ Zhejiang Heze Pharmaceutical Technology Co., Ltd., Hangzhou, China; ^3^ Center for Drug Safety Evaluation and Research, College of Pharmaceutical Sciences, Zhejiang University, Hangzhou, China; ^4^ School of Pharmacy, Hubei University of Chinese Medicine, Wuhan, China

**Keywords:** methyl gallate, NLRP3, uric acid, nephropathy, ROS

## Abstract

Hyperuricemia nephropathy (HN) is a form of chronic tubulointerstitial inflammation, caused by the deposition of monosodium urate crystals (MSU) in the distal collecting duct and medullary interstitium, associated with a secondary inflammatory reaction. Numerous published reports indicated that NLRP3 inflammasome pathway play crucial roles in HN symptoms. The present study aims to investigate the protective effects of methyl gallate on HN mice and the underlying mechanisms. An HN model was established by intraperitoneal injection of potassium oxide (PO) to assess the effect of methyl gallate on renal histopathological changes, renal function, cytokine levels and expressions of NLRP3-related protein in HN mice. Moreover, *in vitro* models of lipopolysaccharide (LPS)-stimulated bone marrow-derived macrophages (BMDMs) and human peripheral blood mononuclear cells (PBMCs) were established to explore the mechanism of methyl gallate on NLRP3 inflammasome activation. The results showed that methyl gallate significantly ameliorated HN by inhibiting uric acid production and promoting uric acid excretion as well as ameliorating renal injury induced by NLRP3 activation. Mechanistically, methyl gallate is a direct NLRP3 inhibitor that inhibits NLRP3 inflammasome activation but has no effect on the activation of AIM2 or NLRC4 inflammasomes in macrophages. Furthermore, methyl gallate inhibited the assembly of NLRP3 inflammasomes by blocking the ROS over-generation and oligomerization of NLRP3. Methyl gallate was also active *ex vivo* against ATP-treated PBMCs and synovial fluid mononuclear cells from patients with gout. In conclusion, methyl gallate has a nephroprotective effect against PO-induced HN through blocking the oligomerization of NLRP3 and then exerting anti-inflammatory activity in the NLRP3-driven diseases.

**Graphical Abstract:**
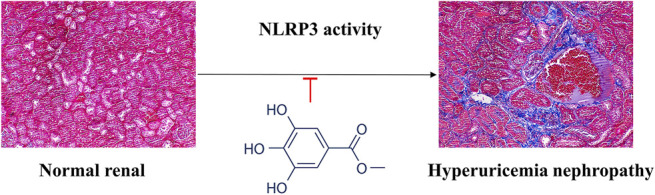


## Introduction

Hyperuricemia is a metabolic disease caused by an imbalance in the synthesis or excretion of uric acid in the body. According to the consensus of Chinese experts on the treatment of hyperuricemia and gout, the incidence of hyperuricemia in China has increased year by year as the improvement of people’s living standard, especially in developed cities and coastal areas, reaching 5–23.5% (Lu et al., 2020). Traditionally, hyperuricemia is thought to induce renal disease through the deposition of urate crystals in the glomerular collecting ducts in a manner similar to that of gouty arthritis (Mazzali et al., 2001; Lu et al., 2019). At present, drugs that have been listed for the treatment of hyperuricemia nephropathy (HN) are mainly including xanthine oxidase (XOD) inhibitors, uricosuric drugs, recombinant uricase supplements and other major classes. However, the widespread clinical use of these drugs are large limited due to the adverse effects and hepatotoxicity (Sattui and Gaffo, 2016). Therefore, it is urgent to explore a drug which has effects on both reducing the circulating uric acid synthesis and increasing renal uric acid excretion in the treatment of HN, so as to ameliorate the renal inflammation, renal fibrosis, oxidative stress or other injuries.

Polyphenols have a wide range of therapeutic effects because of their powerful antioxidant, anti-inflammatory, and immunomodulatory actions, with potential roles on different complications caused by oxidative stress, such as cardiovascular and neurodegenerative diseases (Yahfoufi et al., 2018). Recently, polyphenols have received considerable attention for their potential in modulating immune signaling pathways, inhibiting inflammatory mediators, and regulating various pathological states, including ischemia/reperfusion injury, neurodegenerative diseases, neuropathic pain, and arthritis (Chen et al., 2011; Chen and Lan, 2017; Rahimifard et al., 2017; Yahfoufi et al., 2018; Azam et al., 2019).

Methyl gallate is a gallotannin, widely distributed in edible plants such as *Rosa rugosa, Terminalia chebula* Retz.*, Smilax china* Linnaeus*, Schinus terebinthifolius* Raddi*, Givotia rottleriformis* Griff.*, Bergenia ligulata* (Wall)*,* and *Paeonia sufruticosa* Andr. (Cho et al., 2004; Acharyya et al., 2015; Kamatham et al., 2015; Rosas et al., 2015; Sharanya et al., 2018; Park et al., 2019). Several studies have demonstrate that methyl gallate is associated with significant biological effects of antioxidant (Whang et al., 2005; Crispo et al., 2010), anti-tumor (Lee et al., 2010; Lee et al., 2013), antimicrobial activities (Choi et al., 2009), and anti-inflammatory effect on experimental colitis and arthritis (Kang et al., 2009; Correa et al., 2016). However, the effect of methyl gallate on HN, a condition associated with the development and progression of renal disease due to elevated uric acid levels, is unclear. The present study was aimed to explore the role of methyl gallate on HN and the underlying molecular mechanisms of its anti-inflammatory activity in the activation of NLRP3 inflammasome.

## Materials and Methods

### Materials

Detail of materials are described in the online supplemental file.

### Experimental Animal

Male C57BL/6 mice (No. SCXK (E) 2015-0018) were purchased from Hubei experimental animal research center (Wuhan, China), and *Nlrp3* ^*−/−*^ mice were purchased from Shanghai Model Organisms Center, Inc. The animals were housed in a temperature-controlled, regular 12-h light/dark cycle and provided with standard chow and water *ad libitum*. All experimental procedures were performed in accordance with the regulations of the Animal Ethics Committee of South-Central University for Nationalities (SCUN) (approval number: 2020-scuec-025), all procedures involving animals were performed in accordance with the SCUN Animal Experimentation Guidelines.

### Cell Culture and Stimulation

Bone marrow-derived macrophages (BMDMs) were isolated from bone marrow of 6-8 weeks old mice and cultured in Dulbecco’s modified Eagle medium (DMEM) supplemented with 10% FBS and 1% MCSF for 6–7 days. Human Lymphocyte Separation Medium (catalog no. P8610-200, Solarbio) was used to obtain human peripheral blood mononuclear cells (PBMCs). Prior to stimulation, PBMCs were cultured overnight in RPMI 1640 medium. To activate NLRP3 inflammasome, BMDMs or PBMCs were primed or un-primed with LPS (100 ng/ml, for 3 h) before being stimulated with nigericin (10 μM, for 1 h), or monosodium urate crystals (MSU) (200 μg/ml, for 6 h). To activate the other inflammasomes, salmonella typhimurium or poly A:T was added to the LPS-primed BMDMs.

### Animals and Modeling

Healthy adult male mice fed in clean-grade animal houses with free food and water. After 1 week of adaptive feeding, mice were randomly divided into five groups (*n* = 10 in each group). 1) control group, injected intraperitoneally or given orally with equal volumes of saline; 2) vehicle group, injected intraperitoneally with PO (300 mg/kg) and given orally equal amounts of saline, 3) and 4) methyl gallate groups, injected intraperitoneally with PO (300 mg/kg) and orally given methyl gallate 20 or 40 mg/kg, respectively; 5) allopurinol group, injected intraperitoneally with PO (300 mg/kg) and given orally allopurinol 5 mg/kg. Mice were given saline, methyl gallate or allopurinol by gavage 30 min before PO injection for 28 constitutive days.

### Measurement of Uric Acid, Creatinine and BUN

Serum urate (Sur), urinary urate (Uur), serum creatinine (Scr), urinary creatinine (Ucr) and blood urea nitrogen (BUN) levels were measured using standard diagnostic kits. Protein concentrations were measured by the Bradford method using bovine serum albumin as a standard. Each test was performed in triplicate. The fraction excretion of uric acid (FEUA) was then calculated to assess the uric acid excretion-promoting effect of methyl gallate: FEUA = [(Uur) × (Scr)]/[(Ucr) × (Sur)] × 100, expressed as a percentage (Chen et al., 2011).

### Enzyme-Linked Immunosorbent Assay

IL-6, IL-1β, TNF-a and IL-18 were detected by ELISA kits. The specific method and steps of the experiment were tested according to the experimental guidance given by R&D.

### XOD Activity Detected

The liver tissue was removed from −80°C, 900 μL of pre-cooled physiological saline was added per 100 mg of tissue, homogenized in a grinder, and centrifuged at 8,000 r/min (4°C) for 10 min, the supernatant was obtained, and continue to centrifuge at 15,000 r/min for 30 min, remove the uppermost turbid material, and absorb the supernatant. The protein concentration was measured by BCA method, and the activity of XOD in liver and serum was detected by referring to the kit instructions.

### Reactive Oxygen Species Staining

BMDMs at 2 × 10^5^/ml were placed overnight on coverslips (Thermo Fisher Scientific) in 12-well plates. 12–18 h later, the medium was replaced with Opti-MEM containing 1% FBS. After that, methyl gallate was added for another 0.5 h as described, and then stimulated with MSU. Finally, BMDMs were stained with 2′,7′-dichlorofluorescin diacetate (DCFDA), and then washed with ice-cold PBS for three times and fixed with 4% PFA in PBS for 15 min.

### ROS Assay

BMDMs were cultured in 96-well plates (1 × 10^6^ cells/well) using phenol red-free RPMI medium (Gibco) for 24 h at 37°C in a humidified incubator with 5% CO_2_. Cells were then loaded with the ROS-specific fluorescent probe H2DCFDA (20 μM final concentration; Sigma-Aldrich) for 30 min, washed twice with preheated medium, and exposed to MSU (200 μg/ml). In the treatment group, cells were incubated with 10 μM CP105,696 for 40 min prior to the addition of the MSU. Fluorescence was assessed at 10-min intervals over 1 h using a spectrofluorometer (Synergy 2; BioTek) with a fluorescein isothiocyanate filter (excitation 485 nm, emission 538 nm).

### Hematoxylin and Eosin Staining

The left kidney of the mouse was fixed in 4% paraformaldehyde. After paraffin embedding, 4 μm serial sections were taken and H&E staining was performed by conventional methods.

### Protein Extracts and Western Blotting

The kidney tissue obtained from the experimental mice in each group was first rinsed gently with pre-chilled 1 × PBS buffer twice, then the kidney tissue was placed in 2 ml EP, 500 μL of protein lysis solution was added, and homogenized with a tissue homogenizer. It was then centrifuged at 4°C and 12,000 rpm for 10 min using a low-temperature high-speed centrifuge. After centrifugation, the supernatant was sucked out and placed in a 1.5 ml EP tube and marked on ice. Then the total protein content was detected by BCA kit.

The above protein samples were denatured in Laemmle buffer and then boiled at 95°C for 10 min, separated by SDS-PAGE and transferred to PVDF membranes. Membranes were incubated overnight with primary antibodies (caspase 1, IL-1β and GSDMD lysed forms) and proteins were detected by enhanced chemiluminescence with anti-rabbit secondary antibody or anti-mouse secondary antibody.

### Statistical Analysis

All experiments were performed at least three times. Values are presented as mean ± S.D. When means of two groups were compared, a 2-tailed Student’s t-test was used to determine significance. When multiple treatment groups were compared, one-way ANOVA and Tukey’s post hoc test were used to calculate significance between means. * *p* value < 0.05 was considered statistically significant. Data handling and statistical processing were performed using GraphPad Prism 8.0 (GraphPad Software, San Diego, CA, United States).

## Results

### Effect of Methyl Gallate on Hyperuricemia and Renal Dysfunction

We first investigated whether methyl gallate had the effect on improving renal function and suppressing high uric acid levels in PO-treated mice. According to our previous study, 4 weeks of intraperitoneal injection of PO can lead to HN in mice. Table 1 summarized the anti-hyperuricemia and renal protection effect of methyl gallate. Sur, Scr and BUN levels were significantly increased in the vehicle (PO-treated) mice compared to that of control mice. Methyl gallate at doses of 20 and 40 mg/kg and allopurinol at 5 mg/kg significantly reversed the levels of SUA, SCr and BUN in hyperuricemia mice to the normal value. Moreover, PO treatment could induce an obvious reduction in UUA and UCr levels compared with that of saline-treated mice. Methyl gallate at 20 and 40 mg/kg could significantly elevate UUA and UCr levels in hyperuricemia mice. As previously reported (Hu et al., 2009), FEUA, an important parameter of renal uric acid handling, was significantly reduced in hyperuricemia mice. In the present study, 20 and 40 mg/kg of methyl gallate and allopurinol significantly reversed FEUA in PO-treated HN mice.

**TABLE 1:** The effects of methyl gallate on serum and urinary levels of uric acid and creatinine, as well as FEUA and BUN in PO-induced HN mice.

Group	Dose (mg/kg)	SUA (mg/dl)	SCr (mg/dl)	UUA (mg/dl)	UCr (mg/dl)	BUN (mg/dl)	FEUA
Control	PBS	2.45 ± 0.51	0.87 ± 0.08	43.56 ± 4.93	29.45 ± 3.51	30.20 ± 2.83	54.27 ± 11.25
Vehicle	PBS	5.89 ± 0.77^##^	2.35 ± 0.48^##^	30.68 ± 4.26^##^	30.83 ± 2.66	51.07 ± 6.87^###^	40.74 ± 12.22^##^
AL	5	3.36 ± 0.12^**^	1.68 ± 0.12^**^	34.26 ± 3.55	30.43 ± 3.88	42.35 ± 3.22^*^	57.38 ± 11.09^**^
MG	20	4.26 ± 0.53^*^	1.78 ± 0.21^*^	36.84 ± 4.83^*^	27.08 ± 3.04	40.02 ± 4.06^**^	57.57 ± 11.03^**^
—	40	3.18 ± 0.58^**^	1.44 ± 0.13^***^	40.55 ± 5.12^**^	26.44 ± 4.29^*^	36.82 ± 4.47^***^	72.22 ± 17.81^***^

Uric acid, creatinine and urea nitrogen in serum and urine were detected by kits. Al: allopurinol. MG: methyl gallate. Values are expressed as means ± S.D. (*n* = 10). Statistical differences were calculated by single-factor ANOVA with Tukey HSD test. Compared with control: ^*#*^ *p* < 0.05, ^*##*^ *p* < 0.01, ^*###*^ *p* < 0.001; Compared with vehicle: ^*^ *p* < 0.05, ^**^ *p* < 0.01, ^***^ *p* < 0.001.

**FIGURE 1:**
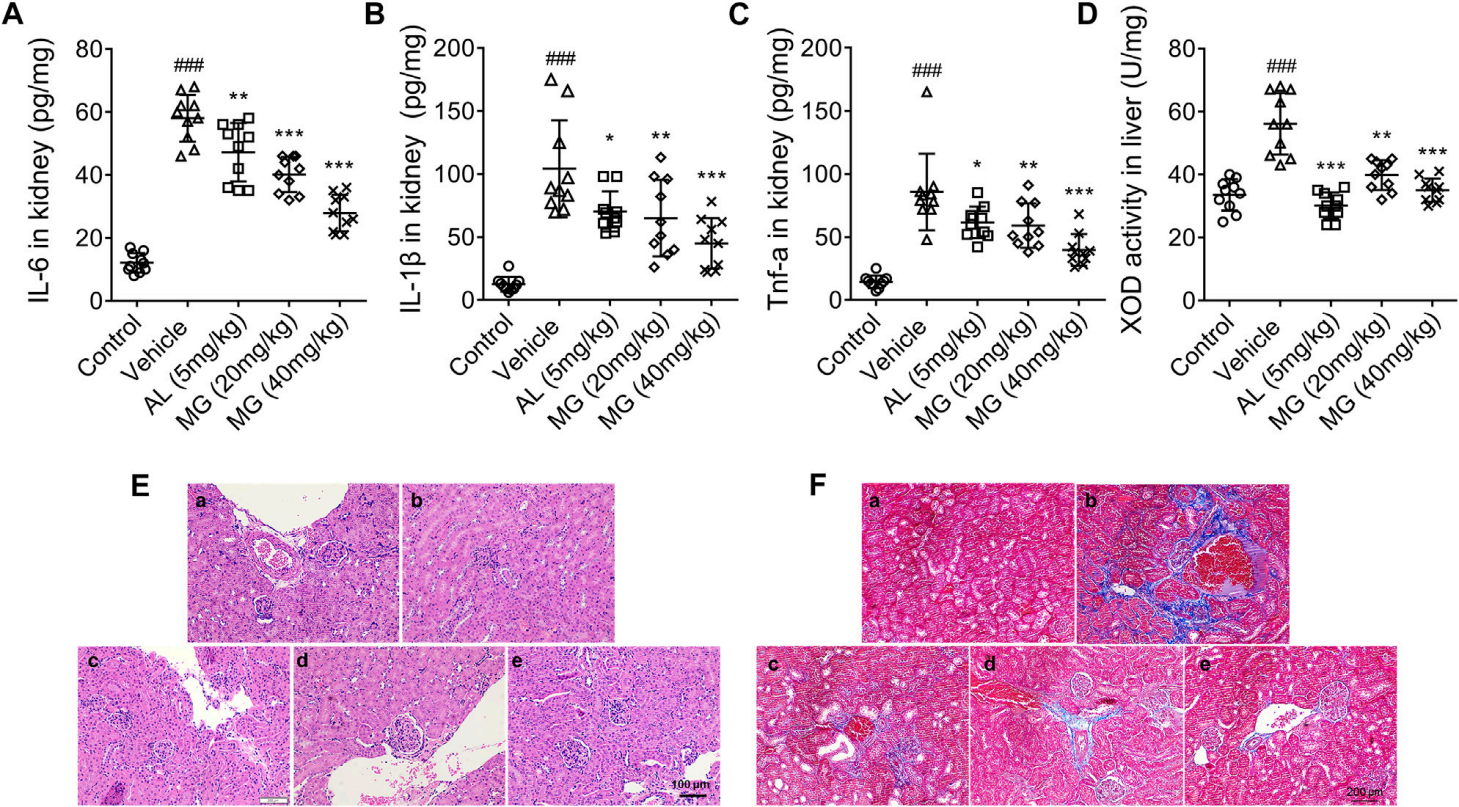
Effect of methyl gallate on PO-induced HN. **(A–C)** Cytokines such as IL-6 **(A)**, IL-1β **(B)** and TNF-α **(C)** in kidney were detected by ELISA kits. **(D)** The activity of XOD in liver was detected by referring to the kit instructions. **(E,F)** HE-staining **(E)** or Masson staining **(F)** of kidney from mice in different groups. Scale bar, 100 μm. a: control; b: vehicle; c: allopurinol (5 mg/kg); d: MG (20 mg/kg); e: MG (40 mg/kg). Values are expressed as means ± S.D. (*n* = 10). Data were analyzed by single-factor ANOVA with Tukey HSD test. Compared with control group: ^*###*^ *p < 0.001*; Compared with vehicle group: ^*^ *p < 0.05,* ^**^ *p < 0.01,* ^***^ *p < 0.001.* MG: methyl gallate.

HN is a form of chronic tubulointerstitial inflammation caused by the deposition of MSU in the distal collecting duct. Therefore, we further investigated whether methyl gallate could inhibit the chronic inflammation. The results showed that the levels of TNF-α, IL-6 and IL-1β were significantly increased in serum and renal tissues of PO-treated mice. However, correspondingly the production of TNF-α, IL-6 and IL-1β was effectively inhibited by methyl gallate and allopurinol (Figures 1A–C). It was speculated that this anti-inflammatory effect derived from their uric acid-lowering effect, especially allopurinol.

In the present study, the effect of methyl gallate on uric acid production was also investigated. XOD is a key enzyme that catalyzes uric acid production. XOD activity in the liver of HN mice was markedly attenuated by methyl gallate (Figure 1D), indicating that methyl gallate reduces uric acid production through inhibiting XOD activity *in vivo*.

HN is a chronic renal tubulointerstitial inflammation with histological changes including arteriosclerosis, glomerulosclerosis and tubulointerstitial fibrosis. As aforementioned results, compared to the control mice, the renal tubules of HN mice were significantly damaged, with severe dilatation of the proximal tubules, formation of casts, and massive detachment and necrosis of the tubular epithelium. All these tubulointerstitial lesions were improved to some extent after treatment with allopurinol and different doses of methyl gallate (Figure 1E). In addition, compared to the control mice, there were significant interstitial fibrous-like changes in the renal tissue of HN mice. However, methyl gallate were effective in alleviating these pathological damages, with only mild fibrous-like changes (Figure 1F).

### Effect of Methyl Gallate on NLRP3 Pathway in Kidney

**FIGURE 2:**
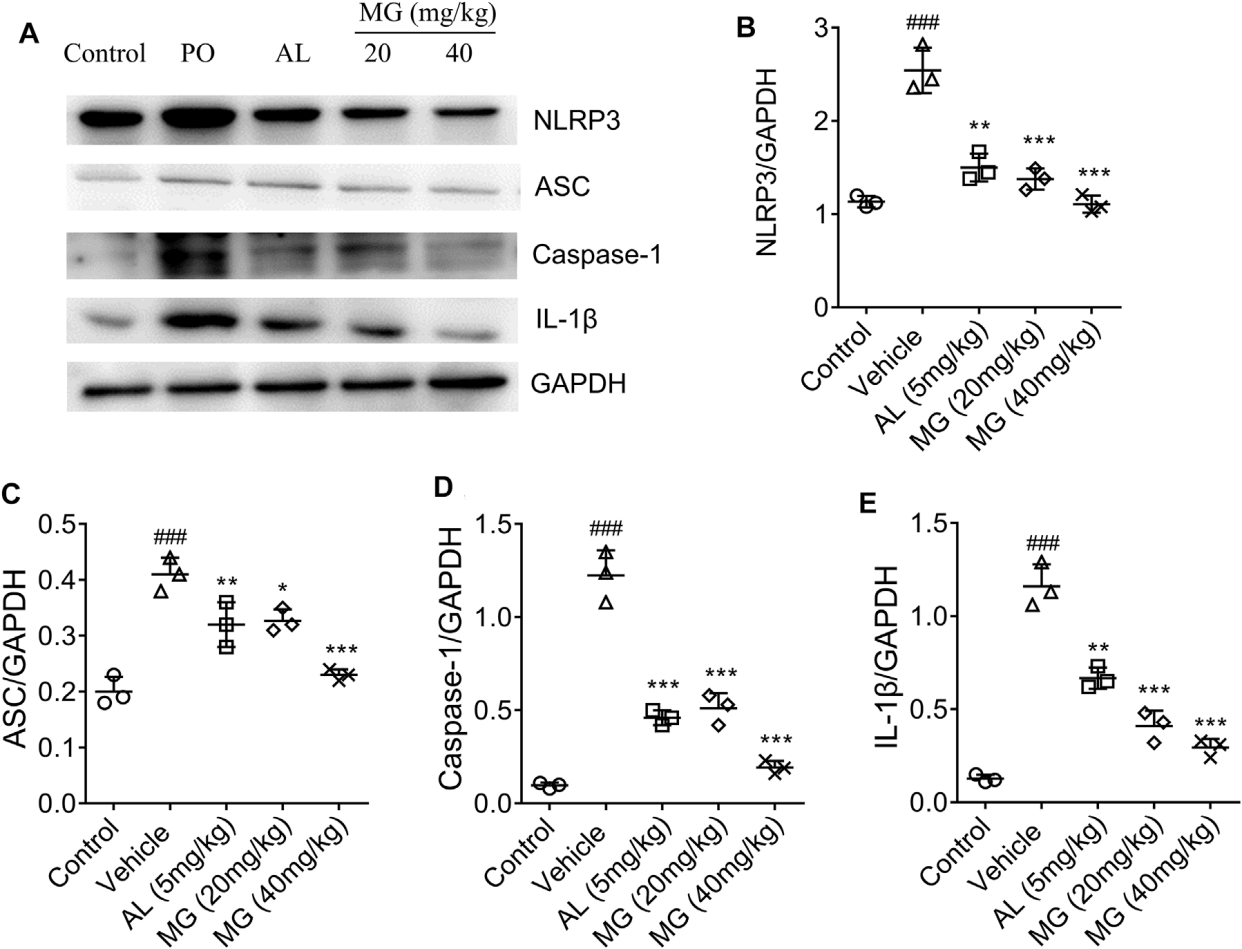
Effect of methyl gallate on NLRP3 pathway in renal tissues. **(A)** Representative western blot analysis of NLRP3, ASC, caspase-1 and IL-1β in renal tissues of mice in different groups. **(B–E)** Western blotting analysis of NLRP3, ASC, caspase-1 and IL-1β in renal tissues of mice in different groups. Values are expressed as means ± S.D. (*n* = 3). Data were analyzed by single-factor ANOVA with Tukey HSD test. Compared with control group: ^###^ *p < 0.001*; Compared with vehicle group: ^*^ *p < 0.05*, ^**^ *p < 0.01.*, ^***^ *p < 0.001.* MG: methyl gallate.

NLRP3 has been reported to sense a variety of stimuli, ranging from bacterial toxins, ATP, crystalline structures such as MSU, silica, and alum (Ghaemi-Oskouie and Shi, 2011; Franklin et al., 2016). The up-regulated expression of NLRP3, ASC, caspase-1 and IL-1β proteins were observed in HN mice, while methyl gallate significantly reversed the levels of NLRP3, ASC, IL-1β and caspase-1 proteins to normal level (Figure 2). These results suggested that methyl gallate had a definite inhibitory effect on NLRP3 inflammasome activity in HN.

### Methyl Gallate Inhibits NLRP3 Inflammasome Activation in BMDMs by ROS Pathway

**FIGURE 3:**
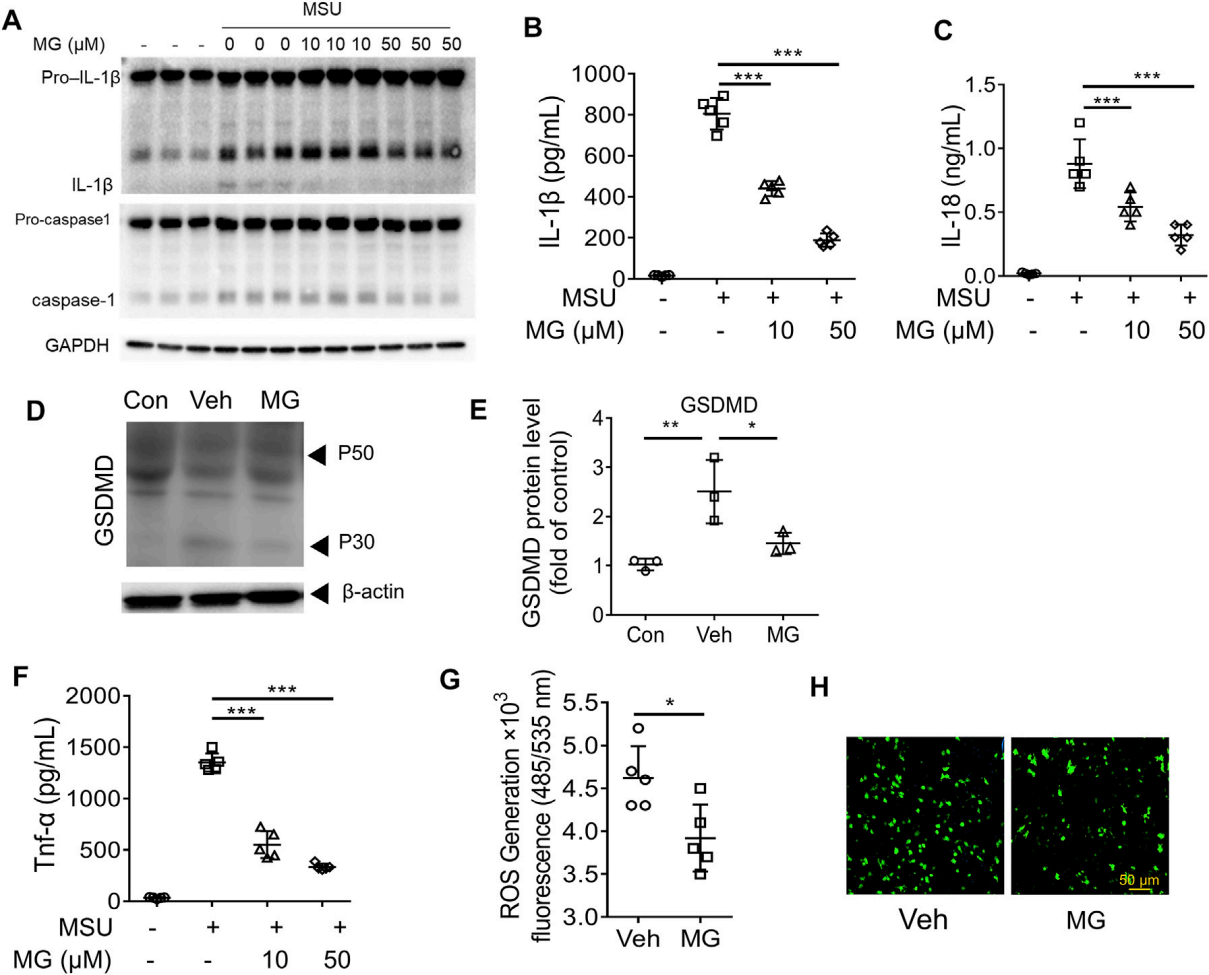
Methyl gallate inhibits NLRP3 inflammasome activation in BMDMs by ROS pathway **(A)** Western blot analysis of the inhibitory effects of MG (10 and 50 µM) on cleaved IL-1β, activated caspase-1 (P20) in culture supernatants (SN) and pro-IL-1β, pro-caspase-1 in lysates (Input) of MSU stimulated BMDMs. **(B,C)** The inhibitory effects of MG (10 and 50 µM) on IL-1β secretion and LDH release in MSU stimulated BMDMs. **(D,E)** Western blot analysis of the inhibitory effects of MG (50 µM) on GSDMD express in MSU stimulated BMDMs. **(F)** The inhibitory effects of MG (10 and 50 µM) on TNF-α secretion in MSU stimulated LPS-primed BMDMs. **(G,H)** The inhibitory effects of MG (50 µM) on ROS production in MSU stimulated BMDMs by ROS assay **(G)** and ROS staining **(H)**. MG: methyl gallate, Veh: Vehicle, Con: Control. Data are expressed as mean ± S.D. (*n* = 6) and are representative of two independent experiments. Statistical differences were calculated by single-factor ANOVA with Tukey HSD test **(B,C,E,F)**, and unpaired Student’s *t*-test **(G)**: ^*^ *p < 0.05*, ^**^ *p < 0.01*, ^***^ *p < 0.001.*

To confirm the mechanism of methyl gallate on inhibiting NLRP3 inflammasome activation, the present study was first investigated whether methyl gallate inhibited caspase-1 cleavage and IL-1β secretion in MSU-stimulated BMDMs. It was observed that methyl gallate treatment blocked MSU-induced caspase-1 cleavage, secretion of IL-1β and IL-18, and pyroptosis in BMDMs (Figures 3A–E). Moreover, methyl gallate was reported to inhibit cytokine-induced activation of NF-κB (Ding et al., 2021; Zhang et al., 2021). In consistent with these studies, methyl gallate significantly inhibits the production of TNF-a in MSU-treated BMDMs (Figure 3F). These results indicated that methyl gallate suppressed both NLRP3 inflammasome activation and related gene expression in MSU-treated BMDMs. It is known that ROS provides the upstream signaling for MSU activation of NLRP3 (Gross et al., 2011), it could be inferred that methyl gallate had an inhibitory effect on ROS overproduction in MSU-treated BMDMs. Indeed, methyl gallate decreased the production of ROS by MSU (Figures 3G,H). This result suggested that methyl gallate was a blocker of the upstream pathway of NLRP3 inflammasome activation.

### Methyl Gallate Specifically Inhibits Activation of NLRP3 by Blocking the Oligomerization of NLRP3 Inflammasome in BMDMs

**FIGURE 4:**
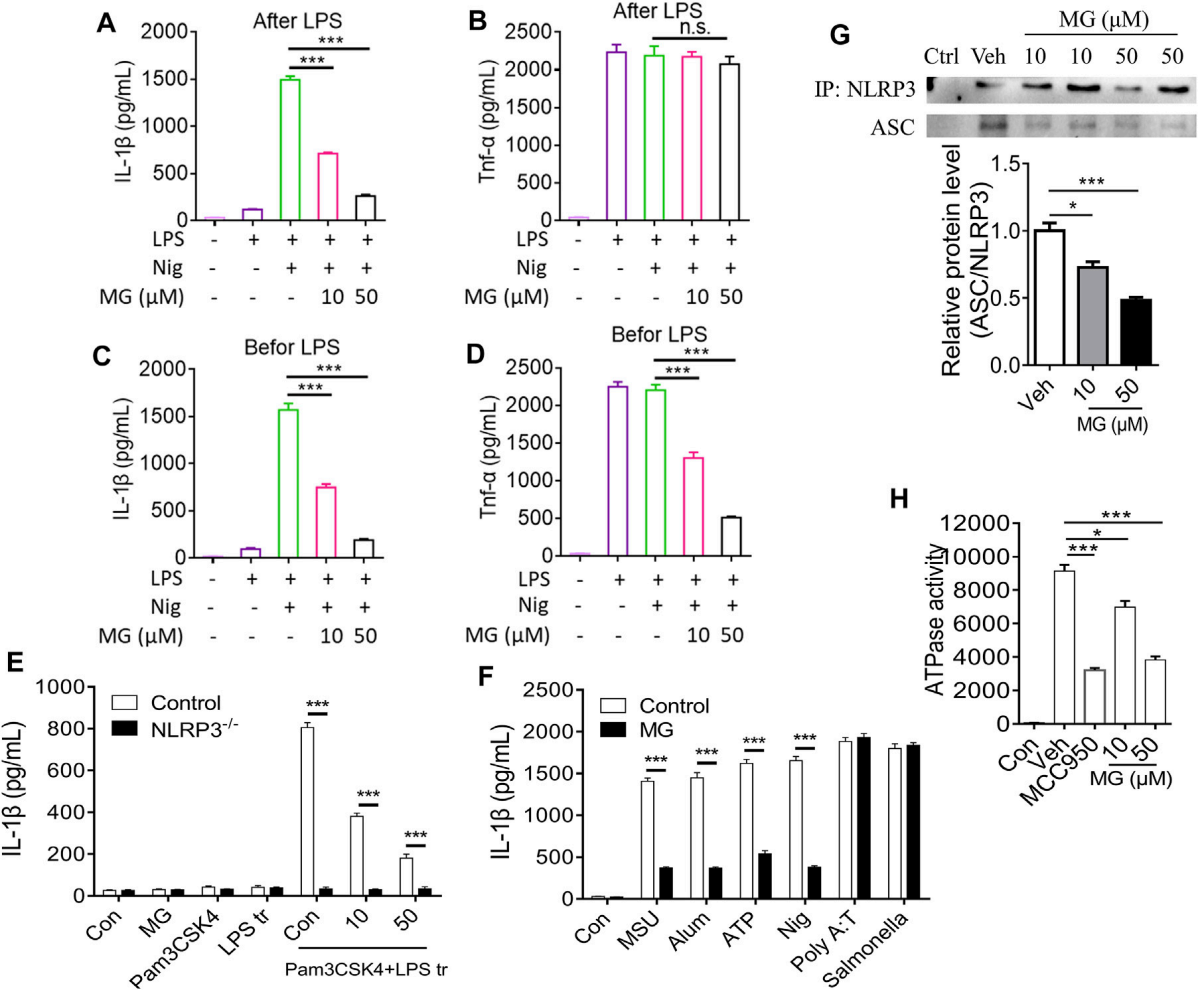
Methyl gallate specifically inhibits activation of NLRP3 by blocking the oligomerization of NLRP3 inflammasome in BMDMs. **(A,B)** BMDMs were treated with different doses of MG for 0.5 h and then stimulated with LPS for 3 h. After that, the cells were stimulated with nigericin. Medium supernatants (SN) were analyzed by ELISA for IL-1β and TNF-α release. **(C,D)** BMDMs were primed with LPS for 3 h and then stimulated with different doses of MG 0.5 h, then the cells were stimulated with nigericin. SN were analyzed by ELISA for IL-1β and TNF-α release. **(E)** The release of IL-1β in BMDMs stimulated with the TLR2/4 agonist Pam3CSK4, and then transfected Wild type and NLRP3 effective cells with LPS. Treatment with MG (10, 50 µM) can reduce the release of IL-1β in BMDMs cells. Cells lacking NLRP3 showed low or no IL-1β production. **(F)** ELISA of IL-1β in SN of LPS-primed BMDMs treated with MG (50 µM) and then stimulated with MSU, nigericin, ATP, Alum, poly A:T and Salmonella. **(G)** Immunoprecipitation (IP, Up) and analysis (Down, mean ± SEM) of the NLRP3-ASC association in BMDMs cells stimulated with LPS/nigericin. **(H)** ATPase activity of recombinant NLRP3 in the presence of MG (*n* = 3), MCC950 (1 μM) (*n* = 3). MG: methyl gallate, Veh: Vehicle, Con: Control. Data are expressed as mean ± S.D. (*n* = 6). Statistical differences were calculated by single-factor ANOVA with Tukey HSD test **(A–D,G,H)**, and unpaired Student’s *t*-test **(E,F)**: ^*^ *p < 0.05*, ^***^ *p < 0.001.*

To further confirm the inhibitory effect of methyl gallate on the activation of inflammasomes, whether methyl gallate could inhibit the secretion of IL-1β in LPS-primed BMDMs treated with nigericin was explored. It was found that methyl gallate treatment blocked nigericin-induced IL-1β secretion but had no effect on the production of TNF-α (Figures 4A,B). In contrast, when BMDMs were incubated with methyl gallate for 30 min before 3-h LPS treatment, methyl gallate inhibited the production of TNF-α (Figures 4C,D). These results further suggested that methyl gallate could suppress the two signaling events of NLRP3 inflammasome activation.

The noncanonical activation of NLRP3 inflammasome by intracellular LPS leads to an alternative inflammasome pathway. We tested the release of interleukin-1β in BMDMs stimulated with the TLR2/4 agonist Pam3CSK4, and then transfected wild type and NLRP3 effective cells with LPS. Treatment with methyl gallate reduced the release of IL-1β in BMDMs cells. Cells lacking NLRP3 showed low or no IL-1β production, confirming the requirement of NLRP3 for IL-1β release (Figure 4E).

In addition to nigericin, we also studied the effects of other NLRP3 agonists (including MSU, Alum, and ATP) to understand whether methyl gallate is a co-inhibitor of NLPR3 inflammasome (Figure 4F). Similar to nigericin, methyl gallate significantly blocked these agonists-induced IL-1β secretion. These results indicated that methyl gallate was a potent and broad inhibitor for NLRP3 inflammasome activation. Additionally, methyl gallate had no effect on the activation of NLRC4 or AIM2 inflammasomes, which were triggered by salmonella typhimurium infection or poly A:T transfection, respectively (Figure 4F). Taken together, these results demonstrated that methyl gallate could specifically inhibit the activation of NLRP3 inflammasomes and the subsequent production of IL-1β.

We next used immunoprecipitation (IP) of the NLRP3-ASC association to study the effect of methyl gallate on the formation of NLRP3 inflammasome in BMDMs stimulated by LPS/nigericin. Following the stimulation of LPS and nigericin, ASC was immunoprecipitated with NLRP3 confirming the oligomer formation (Figure 4G). Methyl gallate led to a reduction in the NLRP3-mediated ASC recruitment (Figure 4G, top and bottom). Using the full-length recombinant human NLRP3 protein, we next gauged the effect of methyl gallate on the nucleotide binding domain of NLRP3. Recombinant NLRP3 exhibited ATPase activity and was inhibited by methyl gallate at 10 and 50 μM (Figure 4H). In the same assay, MCC950, a known inhibitor of the NLRP3 inflammasome, was used as a positive control (Coll et al., 2019). In conclusion, these data suggested that methyl gallate reduced the release of mature IL-1β by blocking the oligomerization of NLRP3 inflammasome.

### Methyl Gallate Inhibits NLRP3-dependent Inflammation *in vivo*

**FIGURE 5:**
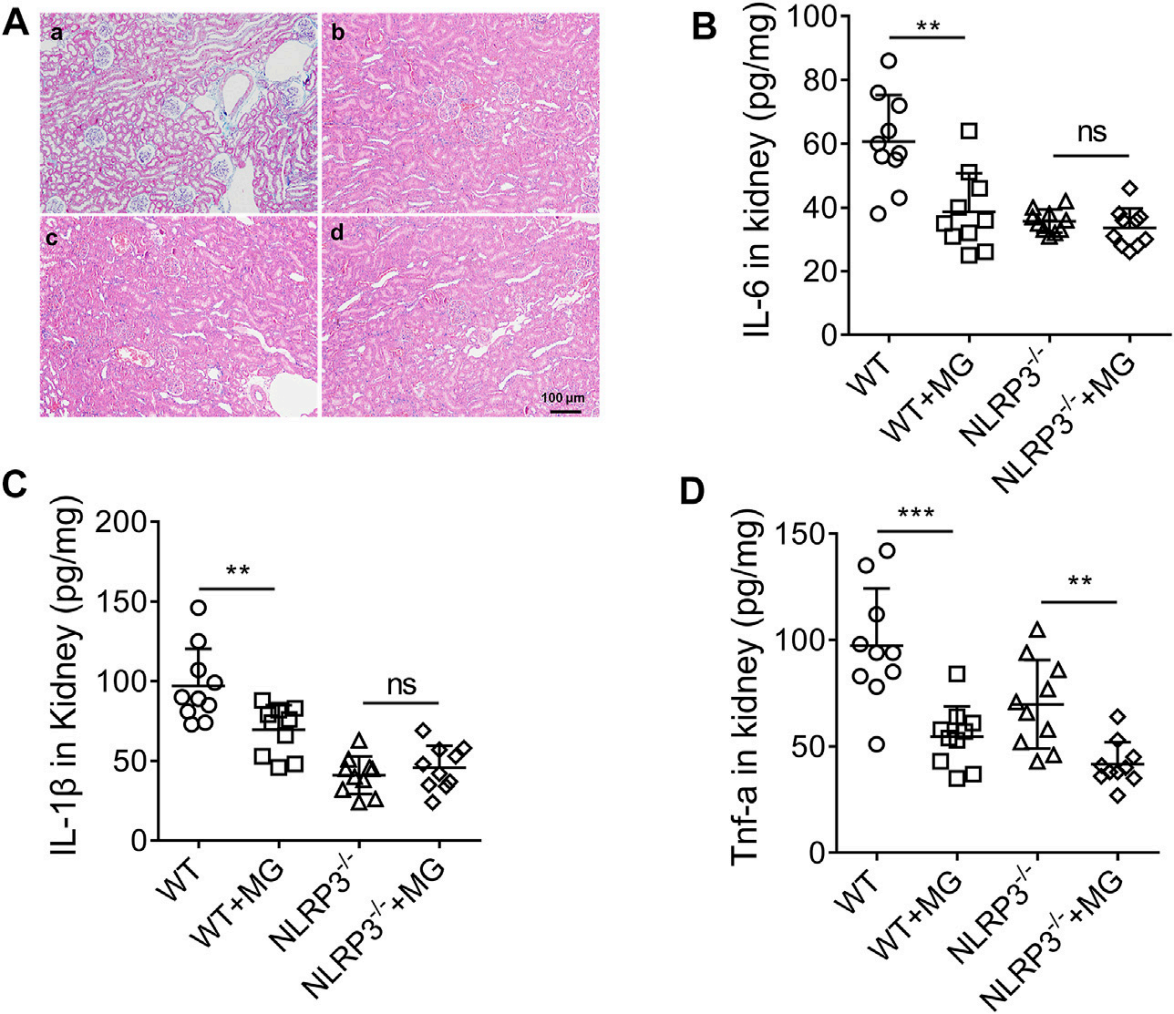
Effect of methyl gallate dependent on NLRP3 pathway in renal tissues. **(A)** HE-staining of renal tissues from mice in different groups. **(B–D)** Cytokines such as IL-6 **(B)**, IL-1β **(C)** and TNF-α **(D)** in renal tissues were detected by ELISA kits. Scale bar, 100 μm. a: WT; b: WT + MG (40 mg/kg); c:*NLRP3* ^*−/−*^; d: *NLRP3* ^*−/−*^ + MG (40 mg/kg). Values are expressed as means ± S.D. (n = 10). Data were analyzed by single-factor ANOVA with Tukey HSD test. Compared with WT or *NLRP3* ^*−/−*^ group: ^**^ *p < 0.01*, ^***^ *p < 0.001*.

We next examined whether methyl gallate could inhibit NLRP3 inflammasome activation *in vivo*. As mentioned above, methyl gallate administration significantly improved PO-induced HN (including renal function, high uric acid levels and FEUA) in wild type C57BL/6 mice, but not in *Nlrp3* ^*−/−*^ mice (Table.2). In addition, methyl gallate administration significantly improved renal histological changes and reduced renal levels of IL-6 and IL-1β in wild type mice but not in *Nlrp3* ^*−/−*^ mice in PO-induced HN (Figures 5A–C). As expected, methyl gallate treatment significantly reduced TNF-a levels in PO-induced wild type mice. In particular, methyl gallate treatment further reduced TNF-a levels in *Nlrp3* ^*−/−*^ mice (Figure 5D). Thus, these findings suggested that methyl gallate protected against inflammation and tissue damage by inhibition of NLRP3 inflammasome.

**TABLE 2:** The effects of methyl gallate on serum and urinary levels of uric acid and creatinine, as well as FEUA and BUN in PO-induced *wt* and *NLRP3* ^*−/−*^ HN mice.

Group	Dose (mg/kg)	SUA (mg/dl)	SCr (mg/dl)	UUA (mg/dl)	UCr (mg/dl)	BUN (mg/dl)	FEUA
WT	PBS	5.24 ± 0.83	2.63 ± 0.32	31.32 ± 3.89	32.15 ± 3.71	49.52 ± 5.86	39.68 ± 8.02
WT + MG	40	2.67 ± 0.54^***^	0.85 ± 0.12^***^	45.63 ± 5.32^**^	27.20 ± 3.95^*^	31.45 ± 4.06^***^	53.66 ± 9.87^***^
*NLRP3* ^*−/−*^	PBS	2.45 ± 0.44^***^	0.83 ± 0.09^***^	44.23 ± 3.87^**^	30.15 ± 4.80	32.98 ± 3.85^***^	55.07 ± 10.14^***^
*NLRP3* ^*−/−*^+MG	40	2.29 ± 0.36^a^	0.79 ± 0.11^a^	41.04 ± 4.62^a^	29.03 ± 3.24^a^	33.05 ± 4.19^a^	54.08 ± 9.33^a^

Uric acid, creatinine and urea nitrogen in serum and urine were detected by kits. MG: methyl gallate. Values are expressed as means ± S.D. (*n* = 10) Statistical differences were calculated by single-factor ANOVA with Tukey HSD test. Compared with WT: ^*^ *p* < 0.05, ^**^ *p* < 0.01, ^***^ *p* < 0.001.

a there was no significant difference compared to *NLRP3* ^*−/−*^ group.

### Methyl Gallate has *in vivo* Activity on Cells of Healthy People or Patients With Gout

**FIGURE 6:**
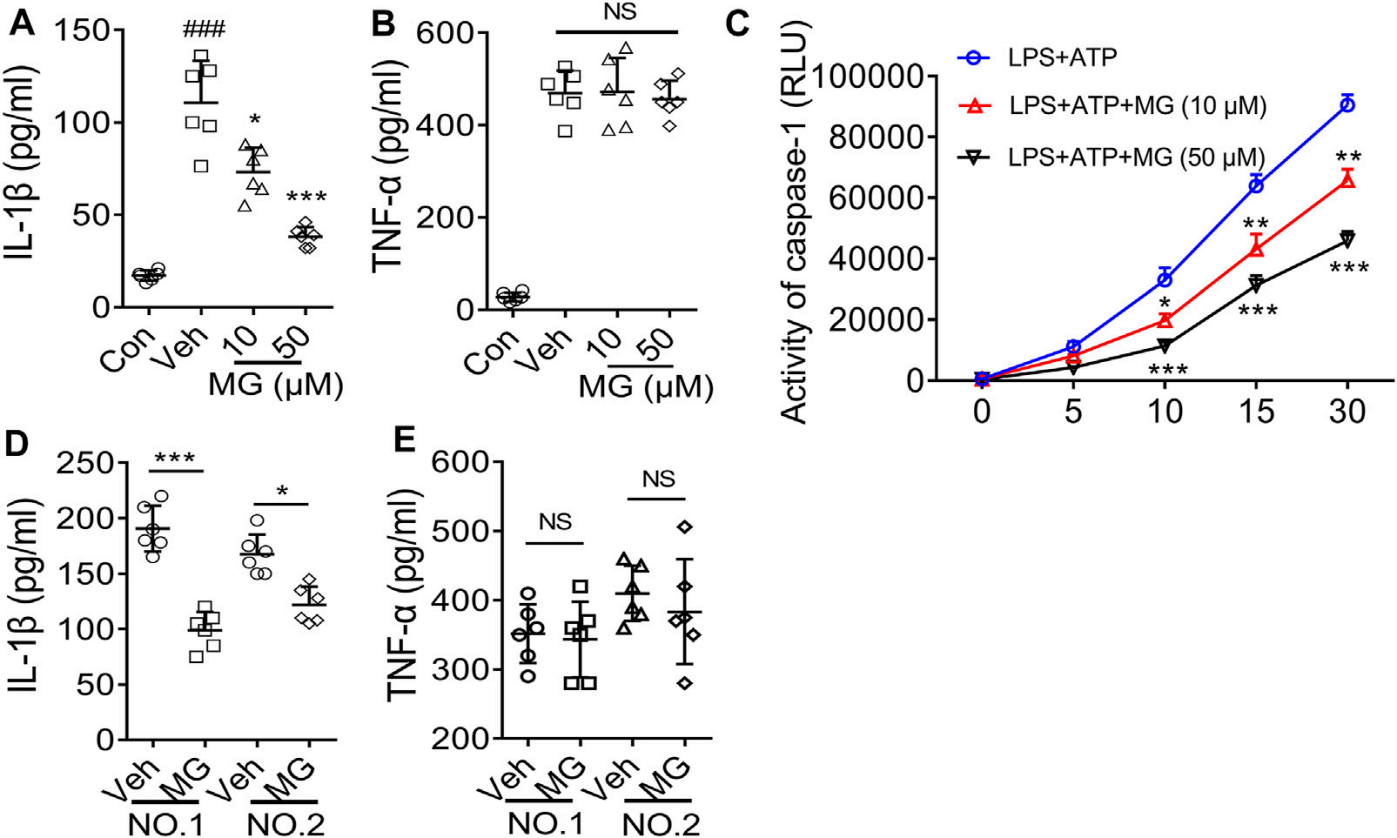
Methyl gallate is active for cells from healthy humans or patients with gout. **(A,B)** ELISA of IL-1β **(A)** or TNF-α **(B)** levels in the supernatants from PBMCs stimulated with LPS and ATP in the presence of concentrations of MG (10 and 50 μM). **(C)** Caspase-1 activity in human PBMCs following LPS + ATP in the presence of MG (10 and 50 μM). **(D,E)** ELISA of IL-1β **(D)** and TNF-α **(E)** in supernatants from SFCs isolated from an individual with gout, treated with MG (50 μM) for 20 h. MG: methyl gallate, Veh: Vehicle, Con: Control. The data are expressed as mean ± S.D. (*n* = 6) of PBMCs from three healthy donors and the SFCs isolated from two patients were analyzed. Statistical differences were calculated by single-factor ANOVA with Tukey HSD test **(A,B)**, or 2-way ANOVA **(C)** with post hoc Sidak test for multiple comparisons, or unpaired Student’s *t*-test **(D,E)**. ^*^ *p < 0.05*, ^***^ *p < 0.001.*

The recruitment of neutrophils and tissue infiltration are hallmarks of the inflammatory response to injury and infection. Thus, we evaluated the effect of methyl gallate on PBMCs after LPS and ATP treatment. As expected, methyl gallate prevented the release of IL-1β (Figure 6A) without affecting the production of TNF-a in PBMCs (Figure 6B). Methyl gallate (10 and 50 μM) also reduced caspase-1 activity in PBMCs (Figure 6C). Therefore, these results suggested that methyl gallate could prevent the activation of NLRP3 in human cells.

Finally, we assessed whether methyl gallate could affect the pre-activated NLRP3 inflammasomes on patient cells with abnormal NLRP3 activation. As expected, IL-1β secretion could be detected in the culture supernatant from synovial fluid cells (SFCs) isolated from two patients with gout arthritis were cultured in the absence of NLRP3 agonist stimulation (Figure 6D). However, when these cells were incubated with methyl gallate (50 μM), IL-1β production were reduced by 65 and 40%, respectively (Figure 6D). In reverse, TNF-α production was unaffected by methyl gallate treatment (Figure 6E). These results suggested that methyl gallate could inhibit the pre-activated NLRP3 inflammasome in patients’ SFCs, supporting the clinical use of methyl gallate for the control of NLRP3-driven disease.

## Discussion

Hyperuricemia refers to the state where the blood uric acid level of adult men is ≥ 420 μmol·L^−1^ and the blood uric acid level of adult women is ≥ 324 μmol·L^−1^ (Dalbeth et al., 2016). Hyperuricemia is closely related to the occurrence of HN and is an important stage in patients with gout (Eleftheriadis et al., 2017). Recent studies have shown that hyperuricemia is an important risk factor for gout, hypertension, diabetes, cardiovascular complications, metabolic syndrome, and kidney disease (Obermayr et al., 2008; Weiner et al., 2008; Johnson et al., 2013). More and more evidence indicate that uric acid-induced inflammation is the core mechanism of hyperuricemia rodent kidney injury (Wang et al., 2015; Chen and Lan, 2017), and the NLRP3 pathway may play a central role in it.

As the incidence of HN has been increasing year by year in recent years, the situation of finding drugs to treat related diseases has become increasingly critical. Most of the drugs available on the market have various adverse effects, such as hypersensitivity syndrome and nephrotoxicity of allopurinol; and hepatic and renal toxicity of benzbromarone. (Lee et al., 2008; Chou et al., 2018). Therefore, the search for an effective drug with few adverse effects is one of the future directions of drug development.

In this experiment, we found methyl gallate could remarkably reduce the uric acid level by inhibit XOD activity in liver. These studies are consistent with previous reports (Masuoka et al., 2006; Asnaashari et al., 2014) that methyl gallate reduced uric acid production by inhibiting XOD activity. Creatinine and urea nitrogen levels are commonly used indicators to evaluate renal function. Methyl gallate could reduce levels of Scr, Ucr and BUN in a dose-dependent manner. Subsequent pathological section results showed that methyl gallate could significantly improve the renal pathological changes (including arteriosclerosis, glomerulosclerosis and tubulointerstitial fibrosis). These results above suggest that methyl gallate indeed improve HN.

Patients with hyperuricemia activate the self-inhibited inactive NLRP3 through MSU as an activation signal that is recognized and bound by the leucine-rich repeat (LRR) of NLRP3 (Kim et al., 2015; Wen et al., 2021). Activated NLRP3 recruit’s apoptosis-associated speck-like protein containing a CARD (ASC) and caspase-1 to form the NLRP3 inflammasome, which cleaves IL-1β precursor proteins and generates IL-1β by regulating caspase-1 activity. At the same time, it induces cell membrane perforation damage, leading to inflammatory death, which facilitates IL-1β release and causes inflammation occurrence (Franchi et al., 2009). In this study, we found that methyl gallate significantly reduced the NLRP3 inflammasome activation (the protein expression levels of NLRP3, ASC, IL-1β, and caspase-1).

Additionally, we demonstrated that MG had dual inhibition of NLRP3 inflammasome activation. As previously reported (Domanski et al., 2016), firstly, methyl gallate inhibits the activation of NF-κB and suppresses the production of TNF-α, an inflammasome-independent cytokine. Secondly, methyl gallate directly inhibited NLRP3 inflammasome activation to decrease IL-1β production. Interestingly, our findings showed that the beneficial effect of methyl gallate on HN was absent in *Nlrp3* ^*−/−*^ mice, indicating that the anti-inflammatory activity of methyl gallate *in vivo* depended on its inhibition of the NLRP3 inflammasome. These findings showed that methyl gallate binds directly to NLRP3 and displayed significant anti-inflammatory activity both *in vitro* and *in vivo*, and thus methyl gallate may have good therapeutic potential for NLRP3-driven diseases. It is believed that although both NLRP3 inflammasome components and upstream signaling events can be targeted, only targeting NLRP3 itself can specifically block its activation (He et al., 2018). Our results indicated that although NLRP3-activated upstream signaling events such as ROS were influenced by methyl gallate processing, we also found that methyl gallate blocked the interaction between NLRP3 and ASC, an important step in NLRP3 inflammasome assembly (He et al., 2016; Shi et al., 2016). Methyl gallate did not inhibit NLRC4 or AIM2 inflammasome activation, which was consistent with previous reports indicating that ASC interacted with NLRP3 but not with other inflammasome sensors, such as NLRC4 and AIM2 (Shi et al., 2016). Thus, our results indicated that methyl gallate was a classical NLRP3 inhibitor.

Consistent with the results from mouse BMDMs, methyl gallate significantly reduced ATP-induced robust inflammasome activation in cultured PBMCs isolated from healthy subjects and inhibit the pre-activated NLRP3 inflammasome in patients’ SFCs. These results indicated that methyl gallate was a specific inhibitor of NLRP3 inflammasome activation in rodent and human innate immune cells.

**FIGURE 7:**
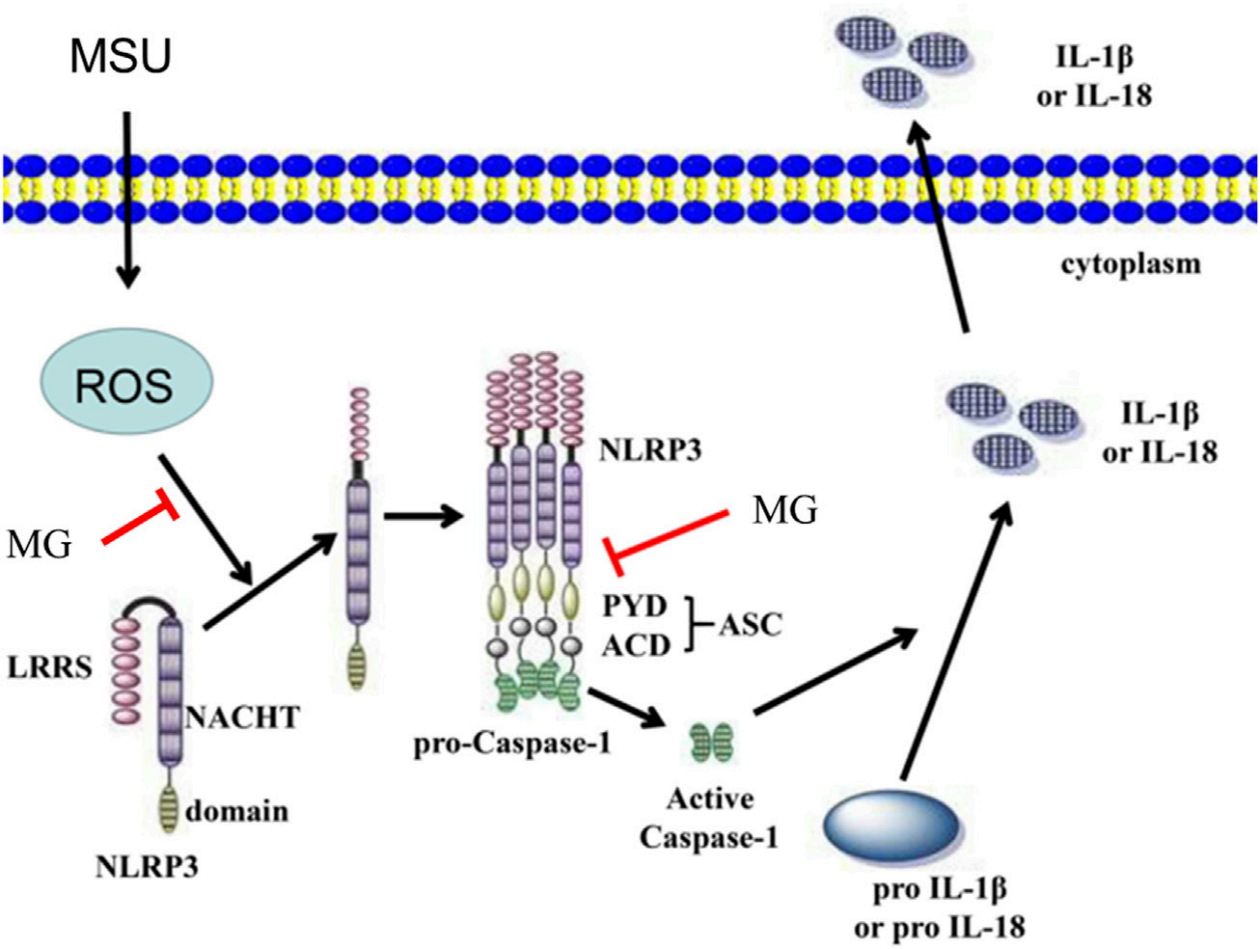
Proposed model for methyl gallate improves PO-induced HN. MG has a protective effect on the kidneys in PO-induced HN. The mechanism may be related to block the overproduction of ROS and prevent NLRP3 inflammasome oligomerization to exert its remarkable anti-inflammatory activity in NLRP3-driven diseases. MG: methyl gallate.

In conclusion, our research provided a potential new and practical drug candidate for the treatment of HN, the mechanism of methyl gallate may involve blocking NLRP3 activation (Figure 7).

## Data Availability

The datasets presented in this study can be found in online repositories. The names of the repository/repositories and accession number(s) can be found in the article/Supplementary Material.
