# Supplementary material for: Methyl Gallate Improves Hyperuricemia Nephropathy Mice Through Inhibiting NLRP3 Pathway
Source: Front Pharmacol. 2021 Dec 20;12:759040. doi: 10.3389/fphar.2021.759040 (PMC8721208; doi:10.3389/fphar.2021.759040)
Supplement: Supplementary file 1 [file Table1.DOCX]

**Supplement Table 1 Reagents and products used in this study**

| **REAGENT or RESOURCE** | **SOURCE** | **IDENTIFIER** |
| --- | --- | --- |
| **Antibodies** | | |
| Anti-mouse GSDMD, Purified | BioLegend | Cat# 939702 |
| Anti-mouse Caspase 1 | Adipogen | Cat# AG-20B-0042 |
| Anti-mouse IL-1β | R&D (Fisher) | Cat# AF-401-SP |
| Anti-mouse NLRP3 | R&D System | Cat# MAB7578 |
| Anti-mouse ASC | Abcam | Cat# ab175449 |
| Anti-mouse GAPDH | Thermo Fisher | Cat# MA1-16757 |
| anti-rabbit secondary antibody | Amersham Pharmacia | Cat# NA934V |
| **Chemicals, Peptides and Recombinant Proteins** | | |
| Allopurinol | Sigma | Cat# 315-30-0 |
| Methyl gallate | Sigma | Cat# 99-24-1 |
| MCC950 | Sigma | Cat# 256373-96-3 |
| Alum | Fisher Scientific | Cat# 77161 |
| Nigericin. sodium salt | Adipogen | Cat# G-CN2-0020-M005 |
| Adenosine 5'-triphosphate | Fisher Scientific | Cat# BP413 |
| Lipopolysaccharide | Sigma | Cat# L2637 |
| CP105,696 | Sigma | Ca# 158081-99-3 |
| MSU crystals | Custom made | N/A |
| Uric acid | Sigma | Cat# U2625 |
| Poly A:T | Sigma | Cat# |
| Salmonella | Sigma | Cat# |
| Pam3CSK4 | Sigma | Cat# 112208-00-1 |
| Human Lymphocyte Separation Medium | Lonza | Cat# 17-829E |
| M-CSF | PeproTech | Cat# 315-02 |
| Red blood cell lysis buffer | Roche | Cat# 11814389001 |
| Bovine serum albumin | Sigma | Cat# B4287 |
| **Critical Commercial Assays** | | |
| TNF-a ELISA Kit (mouse) | R&D System | Cat# MTA00B |
| TNF-a ELISA Kit (human) | R&D System | Cat# DTA00D |
| IL-1β ELISA Kit (mouse) | R&D System | Cat# MLB00C |
| IL-1β ELISA Kit (human) | R&D System | Cat# DLB50 |
| Caspase-1 ELISA Kit (human) | Abcam | Cat# ab219633 |
| Caspase-1 ELISA Kit (mouse) | AdipoGen | Cat# AG-45B-0002-KI01 |
| IL-6 ELISA Kit (mouse) | R&D System | Cat# M6000B |
| IL-18 LISA Kit (mouse) | R&D System | Cat# DY7625-05 |
| ATPase Kit | Sigma | Cat# MAK113 |
| ROS Assay Kit | Abcam | Cat# ab113851 |
| XOD Kit | Nanjing Jiancheng Biological Co., Ltd. | N/A |
| **Experimental Models: Organisms/strains** | | |
| Mouse: *c57bl/6j* | Hubei experimental animal research center | N/A |
| Mouse: *Nlrp3^-/-^* | SMOC | N/A |
| **Software and Algorithms** | | |
| Flow Jo | Tree Star | Version 10 |
| GraphPad Prism | GraphPad Software | Version 6 |
