# Supplementary figures and images for: Methyl Gallate Improves Hyperuricemia Nephropathy Mice Through Inhibiting NLRP3 Pathway
Source: Front Pharmacol. 2021 Dec 20;12:759040. doi: 10.3389/fphar.2021.759040 (PMC8721208; doi:10.3389/fphar.2021.759040)

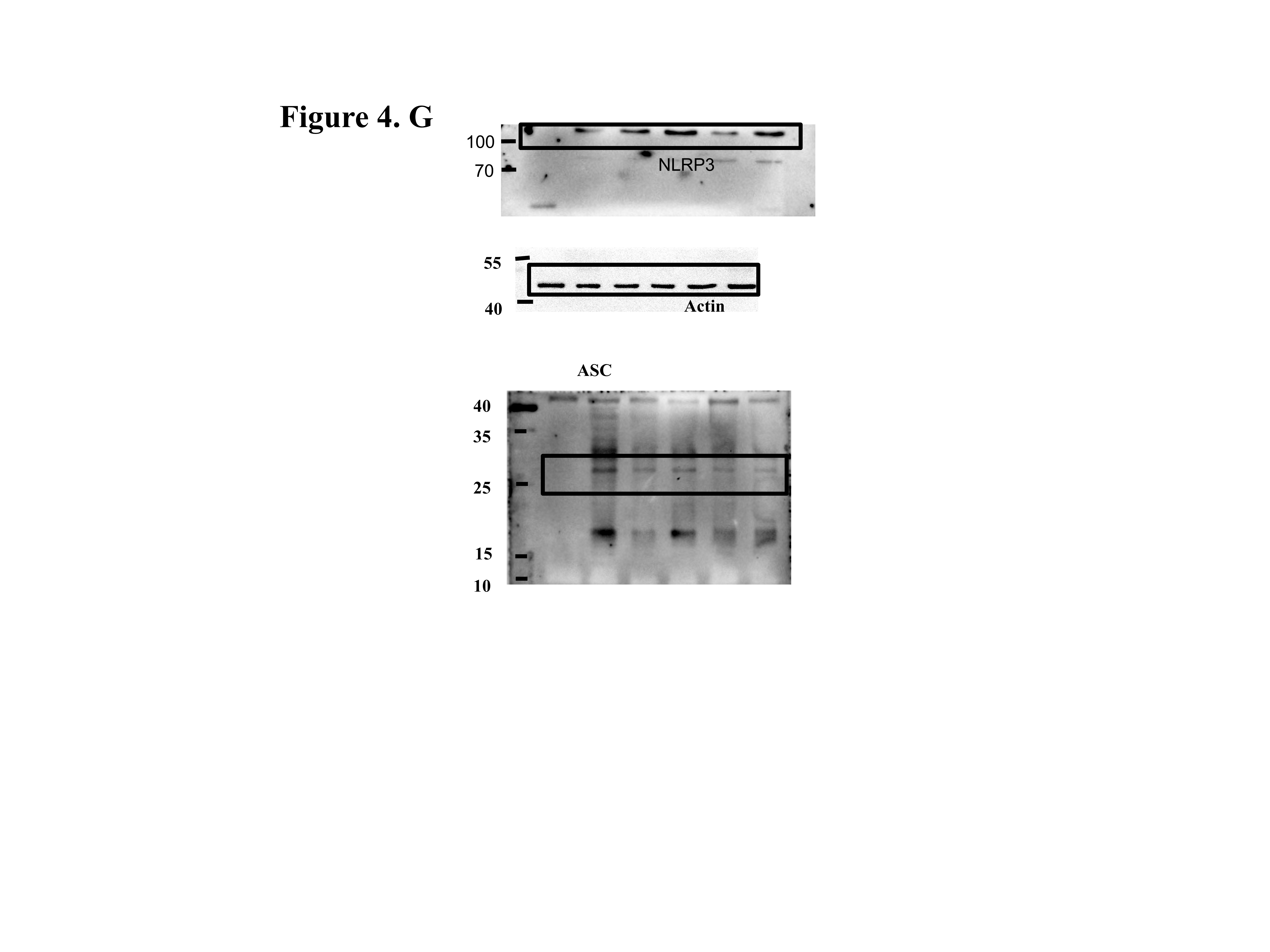

Supplement: Supplementary file 2 [file Image3.TIF]

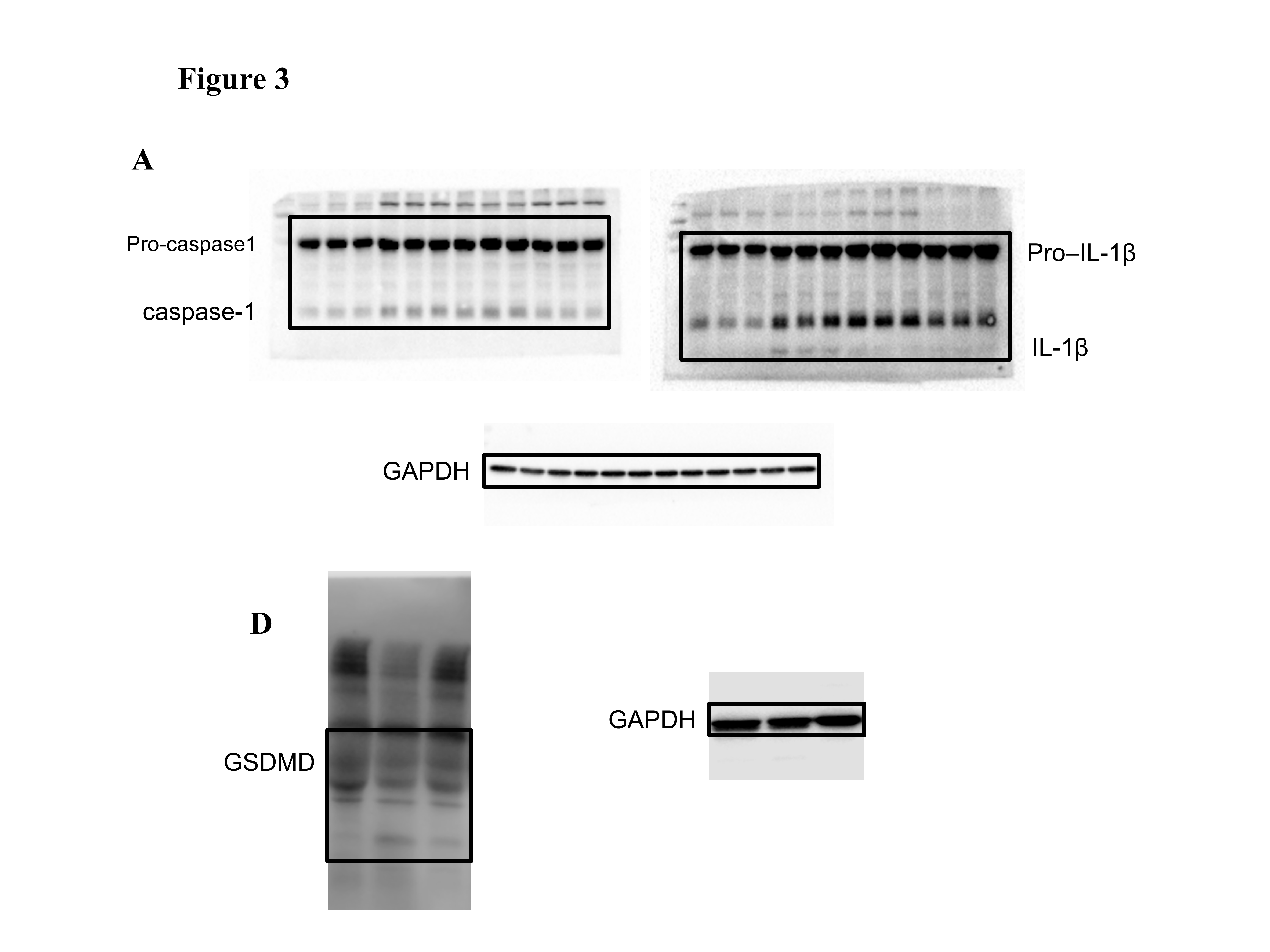

Supplement: Supplementary file 3 [file Image2.TIF]

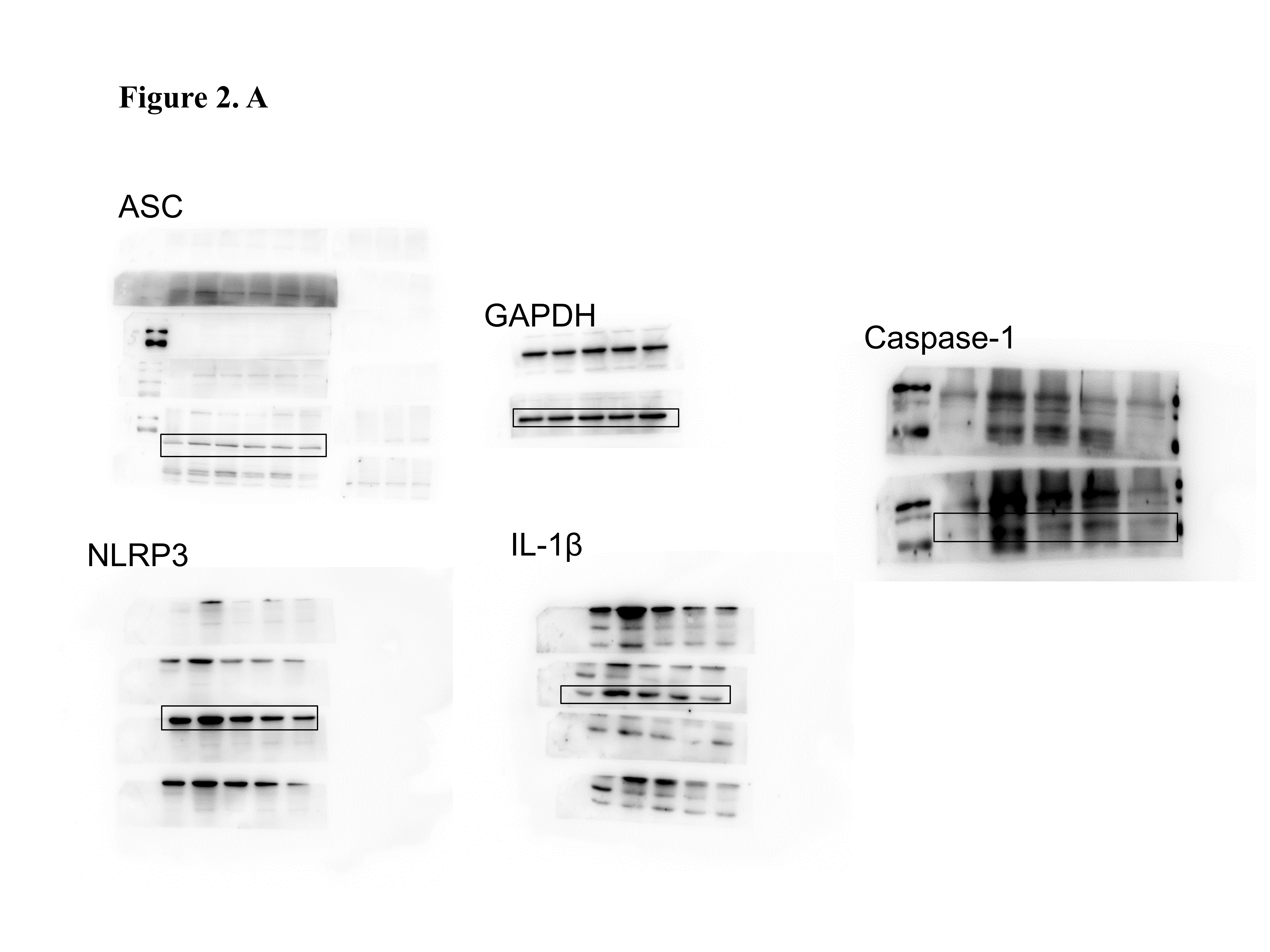

Supplement: Supplementary file 4 [file Image1.TIF]
